# Physiology

**Published:** 1824-09-01

**Authors:** 


*Foreign and Domestic;*

WITH COMMENTARIES.

### I.

### Physiology.

——————— latas a numine leges
Religiosa doce, mentesque cupidine veri
Allice.

1. *Physiological Metamorphoses.** We well remember the time, (some half a century ago) when the transformations of Ovid claimed no small share of our credence. We entertained little doubt that Actæon had been devoured by his own dogs—and it was manifest that the watchful eyes of Argus adorned the gaudy tail of the Peacock. It is true, these airy fictions too soon faded before the light of dull reality—and we had little hope that, in our old age, physiological metamorphoses would rival those of the fanciful Naso. Such, however, is the fact. We have seen the tooth of carnivorous man take root in the head of the grain-eating cock—the "brawny part of Porter's bum" elevated to the loftiest feature of the "human face sublime"—the blood of a sheep circulating in the veins of a citizen—and now, the whole animal creation changing their hides, hairs, and feathers, as readily as harlequin changes dresses in a pantomime. Dr. Dieffenback has been *usefully* employed for many years past in experiments on "*the transplantation of parts of the body from one animal to another.*" Before hazarding any speculation on the grand results which may be expected from these experiments, we shall give a short catalogue of the principal transplantations, referring to Graef's Journal for the details. In the first experiment, the feathers of a *black* chicken were transplanted into the neck, back, tail, &c. of a *white* pigeon. In the second, there was retaliation, as the pigeon's plumes were made to adorn the chicken. In the *third series of experiments*, the feathers of fowls, pigeons, sparrows, &c. were dibbled (by means of

* Experiments on the Transplantation of Animal Substances. By Dr. Dieffenback, of Berlin.

a trochar) into the backs and sides of puppies and rabbits. In the *fourth series*, the bristles of cats and wild rabbits were planted in the skins of pigeons. In the *fifth experiment*, a bunch of feathers was cut from the back of a pigeon, within an inch of the skin—a needle was then pushed down through each stump till the bird shewed symptoms of displeasure—the bristles of a kitten were then introduced into the stumps —took root there, and flourished luxuriously. *Sixthly.* The bristles of a cat and a dog were successfully engrafted on the back of a rabbit. *Seventhly.* The hairs of a friend's eyebrows were transplanted to, and took root in, Dr. Dieffenback's arm. *Eighth Experiment.* A claw from a pigeon's toe was transferred to his tail. It did not stay there, but it deposited an egg—at least a very fine *new* claw sprouted out from the same place. Sometime afterwards the feather which had been plucked out to make room for the claw, grew again, and a furious contest took place between the indigenous and exotic plants—victory at length deciding in favour of the former. *Ninth experiment.* A pigeon's head was scalped, leaving the pericranium on. A flap from the inner side of a pig's thigh, was fitted to the wound, and secured by sutures. It made an excellent scalp, and was soon crowned with a fine grove of bristles. *Tenth experiment.* The nose of a wild rabbit was cut off, and then sewed on again, where it grew as firm as ever.

We do not suppose that any one can be so insensible to the merits of these experiments as to start the rude question—*cui bono?* It is impossible to say what may *not* be the stupendous results to which they may ultimately lead. We shall expect to see the green fat of the turtle transplanted to the thorny back of the skate—and the humble rumps of our barn door fowls sending forth the elegant plumage of the bird of Paradise. The scalping knife of the Indian has lost its terrors. New *scalps* of any size or colour may be readily procured and fitted on, at the nearest friendly *wig-wam.* Lastly, by a very trifling operation, to which the ladies will readily submit, Circassian eye-brows, Grecian noses, and ruby lips, will be as easily and effectually supplied by the experimental physiologist as any other article of head dress by the milliner.

---

2. *Physiology of the Nerves.* In corroboration of the doctrine of Bell and Magendie respecting the nerves of sense and motion, the following physiologico-pathological fact deserves record. A horse became lame of the right hinder extremity on the 27th May, 1823, which lameness disappeared in a few days. He was therefore put to work, but, after half an hour's exercise, he was covered with perspiration,—could hardly support himself on his legs—and soon fell down, his hinder extremities being completely paralytic. But it was found, that sensibility was as entire in these as in the fore legs or other parts of the body. He died the next day, and was carefully examined by M. Buley, veterinary surgeon of Paris. The inferior (corresponding in man to anterior) portion of spinal marrow was found softened and diffluent. The superior (posterior) portion presented no morbid appearance. The pulp of the lumbar and inferior sacral nerves was wanting in consistence—and their envelopes were red and inflamed. We consider this as a very satisfactory confirmation of the doctrine of Mr. Bell.

In an accident which happened to a horse, under the same veterinary surgeon, by which the facial nerve was divided, the curious phenomena and the novel doctrines of Mr. Charles Bell respecting the nerves of the face were also confirmed.

---

3. *Temporary Blindness.* In the practice of Dr. Jones, of Haverford West, a curious case lately took place of blindness during the eruption of modified small pox. This blindness was total, and continued three months, in spite of various remedies. The patient was a little girl, three years of age, who became suddenly affected thus; exhibiting, at the same time, great dilatation of the pupils, "indicating an exudation of lymph in the lateral ventricles of the brain, and consequent pressure on the origin of the optic nerves." At the termination of three months, during which she was otherwise in good health, she began gradually to recover her vision, and the recovery was complete. *Med. and Phys. Jour. April.*

---

4. *Occlusion of the Intestinal and Urinary Passages.* We advise the curators of the college of surgeons to dispatch a confidential agent to the department of the Meuse, in order to treat for the body (dead or alive) of Claude Rouget, more commonly known by the name of DAUDICHE, as a proper companion for the Sicilian dwarf, in the museum. Before we communicate the particulars of this extraordinary case to the public, we must say that we entertain doubts of its authenticity, since the narrator only gathered the items from popular report, or, at best, from the statements of others. At the same time, we do not deny the possibility of the case, though we look for actual ocular demonstration, before we give implicit credence to the narrative.

Dr. Prosper Sylvan Denis, a student in Paris, states that the said *Daudiche* is now sixty-eight years of age—that, till the age of ten, there was nothing particular in his conformation or health—that, at that period, it is said, one of his parents, for some sinister or self-interested purpose, subjected the child to some cruel experiments, by which it would appear that the spine was either broken, dislocated, or contorted, so as to produce lesion of the spinal marrow, paraplegia, and cessation of growth in the lower half of the body. These last phenomena are sufficiently authenticated; but the main wonder is yet to come. The urinary and intestinal canals are obliterated at their terminations, and, consequently, Daudiche never passes either urine or fæces. Yet he eats with a good appetite; but, in a very short time afterwards, vomits up the half-digested aliments, without pain or difficulty—then eats again, and so on.

The lower extremities of this man are no larger than they were at the age of ten years—the sexual organs are almost obliterated—the parietes of the abdomen almost touch the spine, so that there seems a vast vacuum or loss of substance between the ensiform cartilage and pubes. The upper part of the body is sufficiently developed—the memory is prompt and retentive; but the other intellectual faculties, not having been exercised much, cannot be judged of.

Daudiche and his friends resolutely deny all anatomical or physiological examinations; but Dr. Denis states that his father and grand father, both medical men, had opportunities of ascertaining, by personal investigation, the above particulars, and that his statements are principally from their notes. Daudiche, in the quality of mendicant, and mounted on a little stage drawn on wheels, levies a very considerable contribution on the curious and charitable travellers who pass through his native town of Void, on the main road between Paris and Strasburg. He takes his daily station near where the diligences stop, and collects a revenue sufficient for himself and his principal relations. The case is published in the April number of the Archives Generales, and Dr. Breschet, one of the editors, properly remarks that, till death and dissection confirm the above particulars, some doubt must attach to the statement. He cites a case of a similar nature, from Thomas Bartholin, who, while in Italy, saw a man, forty years of age, robust and healthy, who offered no trace of anus or genital organs. This state rendered his sex doubtful, but he was baptized as a female. At the age of twenty-four, however, he shewed a beard, and claimed the privileges of the masculine gender. This man vomited, from time to time, the fæcal remains, through a horn, which he placed in his mouth at those periods, to prevent their contact with the tongue and lips. The urine distilled, *guttatim*, from a small aperture near the umbilicus.

We give these cases as we find them—neither believing with implicit credulity, nor denying with obstinate scepticism, the particular details. Considering the wonder-working powers of nature, there is nothing in the above cases that is calculated to shut out belief in toto. We once saw a female vomit up the remains of her food for more than a fortnight, in consequence of a volvulus—and we verily believe that she died at last, more from the strong medicines we exhibited to force a passage, than from the actual obstruction of the intestinal canal. It is therefore impossible to say, how far nature can compensate for original or early defects in structure or function by vicarious and apparently improbable means.

---

5. *Theory of Fever.* Cullen, Darwin, Brown, and even Broussais, may now hide their diminished heads. The brilliancy of their theories is eclipsed by that which we are going to announce. The remissions and intermissions of fevers have puzzled our best theorists, and no satisfactory solution of the phenomenon has ever been given, *till the present time.* Dr. Bailly (*Bull. des Sc. Med. Mars* 1824) has loosed the gordian knot. His solution rests on the following fact. (Qy, is it a fact?) "All those localities which produce intermittent fevers in man, are those also where epizootic diseases are found among animals. In these last, the symptoms are *continued*—in the former *remittent* or *intermittent*. The post mortem appearances are the same in both. The periodicity of human fevers cannot, therefore, depend on the nature of the agents that produce them, nor upon any difference in the organs affected." What then is the cause?—It is such a brilliant discovery that, really, we are loth to bring it out in the paltry space of a few lines, when it deserves a whole volume for its enunciation. But we are ready to burst with the secret—and out it must come. The cause of the *periodicity* in human fevers, then, and *continuity* in those of brutes, is this: men are perpendicular by day and horizontal by night—whereas, brutes are horizontal by day and by night!!!—"Il a trouvé dans la position toujours horizontale des animaux, et periodiquement horizontale et verticale de l'homme, le moyen de rendre raison de la continuité et de l'intermittence d'affections qui doivent necessairement dépendre de l'uniformité de la circulation chez les uns, et de la periodicité des congestions qui, deux fois chaque jour ont lieu sur nos organes." This discovery has been laid before the INSTITUTE, and we shall soon hear of the accession of "GLOIRE" to French geinus in the report of that distinguished body. God forbid that we should, by a single comment, attempt to detract from the lustre which this discovery is calculated to shed on the genius of continental medicine.

We would just hint, however, *sub rosa*, to Dr. Bailly, that, in Walcheren and Beveland,* where *man* is withered into premature old age by remittents and intermittents, the *cattle* are the finest we ever saw. And, by way of confirming his theory, we would suggest the experiment of causing men to walk on "all fours" in marshy countries, in order to ascertain whether or not they are, iu such positions, liable to the ague.

---

6. *Physiological Experiments.* We began the physiological department of this periscope, in good humour—indeed, somewhat inclined to the merry mood. We could not but laugh at extravaganzas which only went to produce whimsical transformations of Nature, without endangering life or limb of the animals experimented on. We are also well pleased to see experiments made, from time to time, on the inferior animals, for the purpose of elucidating some obscure, or solving some doubtful problem in physiology. But when we observe the whole rising race of candidates for fame rushing impetuously, with knives, needles, saws and poisons, on the living animals around them,—in order to find out some new phenomenon during their torturous experiments, we must pause, and ask, is this the way to clothe the profession in the character of wisdom and humanity as well as science?—we think it is not the way—but our continental brethren are of a very different opinion. In Paris, the mania for vivisections is not repressed, but highly encouraged by institutions which, in other respects, are calculated to further the

* And fifty other places which we could mention.

march of medical science—so true is it that there is no unmixed good in this world. The Royal Institute lauds each successive train of experiments on living animals, no matter how directly contradictory of each other they may be—and thus, a constant stimulus is kept applied to the wild and unbridled ambition, (for it perhaps is not entitled to the name of zeal,) of all ranks of the profession in France. In chemical researches and experiments it is far otherwise than in physiological. *There*, no cruelty is exercised, and every new discovery is almost sure to turn to the advantage of the healing art. How far physiological experiments have contributed to strengthen our hands in therapeutics, we leave to the candour of the most enthusiastic of that party themselves.

We have been led into this train of reflection, on perusing the recent experiments of Magendie, as laid before the Institute, and as repeated before several respectable witnesses in London. Should there be no error or deception in these experiments, (which we are very far from vouching,) we are all at sea respecting the senses. The olfactory are no longer the nerves of smell—the optics of sight—the auditory of hearing! The fifth pair, if they do not exercise all these functions themselves, are, at least, the regents of the senses abovementioned. But we must let M. Magendie tell his own story. It was in the attempt to demonstrate the olfactory properties of the first pair of nerves, that our author found out—or thought he found out—that the sense of smell did not essentially depend on the said nerves.

"1. My first experiment was to lay bare the olfactory nerves in a dog, about a year old. I scarcely expected to find them sensible to the contact of foreign bodies, or even to pricking, the hemispheres of the brain being insensible to such means of irritation throughout the greater part of their mass; and accordingly pressure, pricking them deeply, and tearing them in various ways, produced no effect which indicated sensibility of these nerves. I was curious to see whether the direct contact of a very odoriferous body would afford the same result:—I therefore poured some drops of ammonia upon the nerve; at first the animal seemed not to perceive it, but soon showed proofs of lively sensation. I immediately discovered that the liquid had flowed over the sides of the nerve, had reached its lower surface, and consequently was lodged in the pit of the ethmoid bone. I then was of opinion that the ammonia had acted on the medullary part of the nerve, which we know is expanded upon the cribriform plate, and that at least the white inferior surface of the nerve possessed sensibility, if the cineritious substance of the upper surface did not.

"2. After these observations, I entirely destroyed the olfactory nerves, persuaded that I should then abolish completely the sense of smell. What was my surprise, on examining the animal the following morning, to find that he was yet sensible to powerful odours which I presented to him (such as ammonia, acetic acid, and the essential oil of lavender, &c.) The sensibility of the internal cavity of the nose had lost nothing of its energy: when a probe was introduced, the effects were exactly similar to those in a dog which had not been touched. This extraordinary phenomenon recalled to my memory a fact which I had passed over without much attention last year, because it stood so much opposed to received opinions, that I attributed the occurrence, I know not why, to some fault in the performance of my experiment. I allude to a duck, which survived for eight days after I had removed the hemispheres of the brain, presenting many curious phenomena. Among other singular circumstances, it still retained the power of perceiving strong odours. I exhibited this animal, and subjected it to various proofs of this, in the course I was giving at the time.

" To be better satisfied of the fact, I destroyed the olfactory nerves in many other animals, and the results were exactly similar; but I made the important remark, besides, that the sensibility, which I had before observed at the inferior surface of the olfactory nerve, extended only along the outer border of the cribriform plate; and from this I was led to conjecture, that the sensibility might belong not to the nerve of smell, but to the branch of the ophthalmic which passes from the orbit into the nose through a fissure in the cribriform plate.

" 3. From this suggestion I was led to suspect, further, that the branches of the fifth pair, which are distributed among the cavities of the nose, are the organs by which the sense of smell is maintained after the destruction of the first pair of nerves. These branches in man are pretty numerous, though not very large; they consist, 1st, of the ethmoidal branch of the nasal: 2dly, the naso-palatin branch described by Scarpa: 3dly, of numerous branches which arise from the internal surface of the spheno-palatine ganglion. These nerves coincide in so far that they are all distributed upon the pituitary membrane.

" I was not aware of the exact manner in which the fifth pair gives its branches to the nose of the dog, and I requested M. Desmoulins, who is well skilled in these researches, to dissect this nerve in the dog along with me. We discovered that the ethmoid branch is much larger than in man, and that it supplies a considerable number of small branches to the uppermost part of the nasal cavity; we found besides that there is no spheno-palatine ganglion on the superior maxillary division, but that it sends numerous branches of considerable size, which are distributed on the inferior, lateral, and internal parts of the nose.

" 4. It was then anatomically possible that the whole sensibility of the pituitary membrane might depend upon the divisions of the fifth pair. But conjectures concerning organic functions, derived from anatomy, are worth nothing until they are proved by physiological experiments. I therefore thought of cutting the fifth pair of nerves, in such a manner that the animals might survive. This was more easily said than done. The nerves, as they pass out of the base of the skull, are embraced by the cavernous sinus and the internal carotid artery. Nevertheless I made the attempt in several rabbits; and had the good fortune to succeed in cutting them on both sides, in a number of animals, without producing any very serious injury. I made the same attempt on puppies, kittens, and guinea-pigs, and, when the nerves were once well divided, I was able to assure myself that every trace of the action of powerful odours disappeared. The same animals, which, before the experiment, sneezed or rubbed their noses, or turned away their heads, when they were made to inhale ammonia or acetic acid, &c., continued perfectly undisturbed, when the fifth pair was divided, or only showed symptoms of the odour having affected the larynx. *The result of this experiment, in counterproof of the preceding, appears to me to shew, that the sense of smell, as far as regards powerful odours, depends upon the branches of the fifth pair: and that the first pair of nerves has no share with the fifth in bestowing this function.*

" 5. Here an objection presents itself. The odours employed, it may be said, are very active: they produce a chemical action upon the pituitary membrane just as when they come in contact with the conjunctiva. Is it not possible, when you destroy the sense of touch in the membrane of the nose, that you deprive it, not of the power of perceiving odours, properly so called, but rather of its sensibility to the impression of pungent and caustic vapours; such as those of ammonia and acetic acid? This remark may apply to these two vapours, but not to the oil of lavender or of dippel. At any rate, before the performance of these experiments, it could scarcely have been presumed, that irritating vapours did not act on the sense of smell.

"6. For the purpose of doing away with this difficulty experimentally, I destroyed, by bruising them, (en les broyant) the olfactory nerves in a setter, the fineness of whose nose is well known; and I observed just as in my former experiments, that he could easily perceive powerful odours. But I was desirous of ascertaining whether he could distinguish the smell of meat, or cheese, or food in general. With this intention I enclosed portions of these in paper, and placed them before him; he invariably tore off the paper, and then devoured the contents. Yet I cannot regard this experiment as satisfactory, because the dog, in other circumstances, did not appear able to detect, by its smell, food placed near him without his knowledge. Supposing this last result to be correct, it does not prove, however, that the fifth pair is not the agent of the sense of smell, because the injury which is necessary to destroy the olfactory nerves, necessarily produces inflammation within the cavity of the nose, quite sufficient to injure, in a secondary way, the power of smelling. This subject I am pursuing at present. In chickens, ducks, and magpies, I removed the lobes of the brain with the whole of the olfactory nerves. They continued to possess all the sensibility which belongs to the membrane of the nose, and manifested evident signs of the action of powerful odours on the sense of smell: I cannot comprehend how the contrary has been lately published.

"7. To M. Ramon, medical inspector of the Maison Royale at Charenton, I am indebted for a fact which appears to me to prove, that the integrity of the hemispheres of the brain is not indispensable for the exercise of smelling. It is common to see patients, who have been afflicted for several years with madness, fall suddenly into a condition of dullness and torpidity analogous to complete drunkenness; their limbs totter, their motions are not under their control, and their speech is disturbed; this state, which is beyond the power of relief, is followed by an entire loss of the intellectual faculties, and not long afterwards by death. On examination, the hemispheres are found gorged with blood, the membranes inflamed, and the cortical substance deeply altered. M. Ramon has observed in such individuals that the sense of smell continued, not only with regard to powerful and pungent odours, but also to such as are much more transitory.

"Such are the observations concerning the nerve of smell which I present to physiologists: they are as yet incomplete, and require to be followed up. I hope, however, that they may induce others to repeat them, and not to neglect an opportunity of confirming or invalidating them, by pathological observations.

"8. From these researches it likewise follows, that animals, such as the dolphin, in which there are no olfactory nerves, are probably not deprived of the sense of smell, notwithstanding what has been asserted by naturalists. If it be confirmed that the faculty of smelling belongs to the fifth pair, we have still to enquire, what can be the use of the olfactory nerves and their lobes. Nothing hitherto is known which is likely to point out the way to us, in which case they would require to be added to the list of those parts of the nervous system concerning the functions of which we are still entirely ignorant."* 85.

---

* Vide Med. and Phys. Journal for July.

The next train of experiments which M. Magendie instituted, were on the influence of the fifth pair of nerves on the nutrition and functions of the eye.

After dividing the fifth pair of nerves, within the cranium of a rabbit, our author found that all sensibility was lost on the same side of the face, the inside of the nose, the surface of the conjunctiva, &c. Even ammonia applied to the eye made no impression, while in the other eye it produced violent lachrymation. On the side of the divided nerve the eye was dry, there was no winking of the eyelids, the ball of the eye seemed to have lost all motion, the iris being strongly contracted and immobile. Next day, when the animal was examined, the sound eye was found inflamed by the ammonia, the other presenting no phenomena of the kind. "The section of the nerves had thus prevented the developement of inflammatory action." To enable him to study these various phenomena with care, he that day cut the fifth pair in a number of rabbits—in some, on one side—in others, on both. It was by observing these animals during the following days that he was led to discover the facts now to be stated.

"Twenty-four hours after the division, the cornea begins to become opaque, at the end of seventy-two hours it is much more so,—the opacity encreases, and five or six days after the operation it is as white as alabaster.

"From the second day the conjunctiva becomes red, appears inflamed, and secretes a very abundant, milky, puriform matter: the eyelids are either wide open and motionless, or else they are sealed by the puriform matters which have dried between their edges, and when they are separated a considerable quantity of matter above described escapes.

"Towards the second day after the section, the iris is likewise observed to become red, its vessels are developed, and, in fine, the organ inflames.—False membranes are formed on its anterior surface, which, like the iris itself, have the appearance of a disk pierced in the middle. These adventitious membranes at length fill the anterior chamber and contribute to the opacity of the cornea. Is it not a very extraordinary phenomenon to witness an active inflammation with suppuration, and complete insensibility of the part inflamed, which are caused by the division of a nerve? Before going farther, I would mention that this rapid opacity of the cornea at first appeared to me to depend upon the prolonged contact of the air. To ascertain this, I cut the seventh pair in a rabbit, which, according to Mr. Charles Bell, governs the movement of winking: but, although the eye of this animal remained constantly exposed to the air for many days, no opacity of the cornea took place; nor any inflammation either of the conjunctiva or of the iris. I then suspected that the opacity depended on the want of the secretion of tears. It is possible, said I, that a membrane such as the cornea may have occasion to be constantly soaked by a limpid fluid to maintain its transparency. To ascertain if my conjecture had any foundation, I completely removed the lachrymal gland in two rabbits, but no opacity of the cornea was perceptible during eight days that they survived this extraction. My supposition therefore was without foundation. The opacity of the cornea, the inflammation, and the suppuration of the conjunctiva and of the iris, were thus found to depend upon nervous influence.

"Towards the eighth day after the section of the fifth pair the iris becomes visibly altered; it detaches itself from the sclerotica, and its centre ulcerates: at the end of two or three days the humours of the eye, being muddy and partly opaque, make their escape, and the eye is reduced to a small tubercle, which only occupies a very small part of the orbit, giving something hideous to the aspect of these animals. If the eye be now dissected, it is found to contain nothing but a matter which resembles cheese newly coagulated, and that the retina has almost entirely disappeared; a trace of it here and there is all that can be seen.

"Vision appears to be, if not entirely lost by the division of the nerve, at least very much weakened, and if, some hours after the operation, a sharp instrument be pushed against the surface of the retina, the animal gives no mark of sensibility;—when both nerves are cut in an animal, he appears blind, and his gait is very singular: he only moves with the chin strongly pressed against the ground, pushing his head before him, and using it as a guide in the same way as a blind man does his staff. The conduct of an animal in this state differs entirely from that of one simply deprived of sight, the latter easily directing itself by means of the moustaches and of the sensibility of the skin of the face: he stops before holes, perceives obstacles,—in short, it would often be difficult to know whether he was blind or not. Whilst the animal in whom the fifth pair has been divided has only one way of moving, and instead of avoiding obstacles, often persists in pushing against them for many hours so as to excoriate the skin on the fore part of the head.

"The tongue is insensible on the side where the nerve is cut, and on either side if both nerves be divided. The animal in this case holds it out of its mouth, but is able to draw it in towards the pharynx. Sapid bodies have no apparent action on the anterior part of the organ, but they have an evident effect upon its centre and root. In dogs and cats, the lower jaw drops, after the intersection of the fifth pair on both sides, which greatly impedes deglutition, and sometimes renders it impossible. They have the same gait as rabbits, but instead of resting on the chin, they often press against the tongue which gets underneath, in consequence of the dropping of the jaw, and is rubbed against the ground during progression.

"When only one nerve is cut, changes take place in the nostrils, mouth, and surface of the larynx of the same side: half of the tongue becomes white, the epidermis becomes thickened, the gums separate from the teeth, and food lodges in the intervals thus formed; probably the animals, being no longer arrested by feeling these substances, push them between the teeth and gums without being sensible of it.

"Observation has led me to believe, *that the division of the fifth pair likewise entails the loss of hearing*: this would be less extraordinary, as in many animals the nerve of hearing is evidently only a branch of the trifacial. *If this last result be correct, all the senses would thus be under the influence of the fifth pair, and the general theory of sensations would require to be reformed*"* 88.

The fallacy of *experience* has been acknowledged since the days of Hippocrates, and the observation must apply still more strongly to *experiments* than to experience. In M. Magendie's experiments, we think the sources of error were numerous and prolific. When we consider that, to divide the fifth pair of nerves, the operator was obliged to thrust a sharp cutting instrument through the cranium, the middle lobe of the brain, and almost to the centre of the basis cranii, where, in most instances, M. Magendie himself opened the cavernous sinus, while dividing the

* Vide Med. and Phys. Journal, No. 305.

fifth pair, so that the animals soon bled to death:—when we consider these circumstances, we say, how can it be averred that no injury is done to vitiate the experiments? The third and fourth nerves too, lie in such immediate proximity to the fifth, that, although they may not be actually divided, it is highly probable they receive injury, and that thus their functions are disturbed.

But the paramount source of error evidently lies in the confounding common or general, with specific sensibility. Thus the experimenter tells us, that, after destroying the olfactory nerves, the animal was still sensible to powerful odours, as ammonia, &c. This only proves that common sensibility was present, and warned the animal to avoid disagreeable irritation. His own experiment of the dog and the cheese proves this, though in a different manner. The dog tore off the paper that surrounded the cheese, *when placed in his view*, and devoured the latter. This was, or might be, at least, from *sight*, not smell. When the same viands were placed near him, but not immediately in his view, he intimated no consciousness of their presence. In short, we place little confidence in the truth of M. Magendie's conclusions, though we have considered it our duty to lay his experiments before our readers.

